# Social Isolation-Induced Aggression Potentiates Anxiety and Depressive-Like Behavior in Male Mice Subjected to Unpredictable Chronic Mild Stress

**DOI:** 10.1371/journal.pone.0020955

**Published:** 2011-06-17

**Authors:** Xian-cang Ma, Dong Jiang, Wen-hui Jiang, Fen Wang, Min Jia, Jin Wu, Kenji Hashimoto, Yong-hui Dang, Cheng-ge Gao

**Affiliations:** 1 Department of Psychiatry, First Affiliated Hospital of Medical College of Xi'an Jiaotong University, Xian, China; 2 Division of Clinical Neuroscience, Chiba University Center for Forensic Mental Health, Chiba, Japan; 3 Key Laboratory of Environment and Genes Related to Diseases of the Education Ministry, Key Laboratory of the Health Ministry for Forensic Medicine, Department of Forensic Medicine, Xi'an Jiaotong University School of Medicine, Xi'an, China; Rikagaku Kenkyūsho Brain Science Institute, Japan

## Abstract

**Background:**

Accumulating epidemiological evidence shows that life event stressors are major vulnerability factors for psychiatric diseases such as major depression. It is also well known that social isolation in male mice results in aggressive behavior. However, it is not known how social isolation-induced aggression affects anxiety and depressive-like behavior in isolated male mice subjected to unpredictable chronic mild stress (CMS), an animal model of depression.

**Methodology/Principal Findings:**

C57/B6 male mice were divided into 3 groups; non-stressed controls, in Group I; isolated mice subjected to the CMS protocol in Group II and aggression by physical contact in socially isolated mice subjected to the CMS protocol in Group III. In the sucrose intake test, ingestion of a 1% sucrose solution by mice in Groups II and III was significantly lower than in Group I. Furthermore, intake of this solution in Group III mice was significantly lower than in Group II mice. In the open field test, mice in Group III, showed reduced locomotor activity and reduced entry and retention time in the central zone, compared to Groups I and II mice. Moreover, the distances moved in 1 hour by Group III mice did not differ between night and morning. In the light/black box test, Groups II and III animals spent significantly less time in the light box compared to Group I animals. In the tail suspension test (TST) and forced swimming test (FST), the immobility times of Group II and Group III mice were significantly longer than in Group I mice. In addition, immobility times in the FST were significantly longer in Group III than in Group II mice.

**Conclusions/Significance:**

These findings show that social isolation-induced aggression could potentiate anxiety and depressive -like behaviors in isolated male mice subjected to CMS.

## Introduction

Major depressive disorder, also called major depression, is a debilitating and recurring psychiatric disorder, with a worldwide prevalence of approximately17% [1]. An epidemiological survey carried out from 2001 to 2005, of 113 million adults from four provinces in China demonstrated a 6% prevalence rate for depression [2]. Yet, despite this high prevalence, the pathogenesis of this disorder is not yet fully understood. It has been suggested that stress and altered monoamine, hypothalamic-pituitary-adrenal (HPA) axis, brain-derived neurotrophic factor (BDNF), and glutamatergic neurotransmission might be implicated in the pathogenesis of major depression [3]–[13].

A large body of epidemiological evidence shows that life event stressors are major vulnerability factors for depression [14]–[16]. Furthermore, a relationship between marital status, psychological distress and major depression has been suggested [17]. Longitudinal, community-based data from the New Heaven Epidemiologic Catchment Area program demonstrated that marital disruption was associated with higher prevalence rates of major depression in men [18], suggesting that this type of life event stressor conferred a high risk of disease for men.

Several types of stress, including forced swimming test (FST), tail suspension test (TST), learned helplessness (LH), unpredictable chronic mild stress (CMS), and early life stress, have been used in preclinical models of depression [19], [20]. The CMS model was originally established by Katz [21], [22], and modified by Willner [23]. In the CMS paradigm, rodents are exposed to a variety of relatively mild stresses (e.g., isolation housing, disruption of light-dark cycles, brief food or water deprivation, tilting of home cages) intermittently for relatively prolonged periods of time (e.g., several weeks) [24]–[26]. In rodents, the unpredictable CMS paradigm produced anhedonia - the loss of interest in normally pleasurable and rewarding activities, which is a core symptom of depression [24], [27]–[29]. Furthermore, the CMS paradigm induces various long-term behavioral, neurochemical, neuroimmune and neuroendocrine alterations that resemble those observed in patients with depression, where symptoms are reversed only by chronic, but not acute, treatment with broad spectrum antidepressants [20], [25], [26]. However, the reliability of the CMS model is under question [20], [26], [30].

In contrast, long-term social isolation is a model to study the behavioral and neurochemical consequences of depriving rodents of social interaction. Many of the symptoms caused by long-term isolation resemble those seen in depression and anxiety disorders [31], [32]. Furthermore, long-term isolation of male mice is known to induce offensive and aggressive behavior, such as attacks [31], [33]. Taken together, it is of great interest to examine the effects of social isolation-induced aggression on the anxiety and depressive-like behavior in male mice previously subjected to CMS.

**Figure 1:**
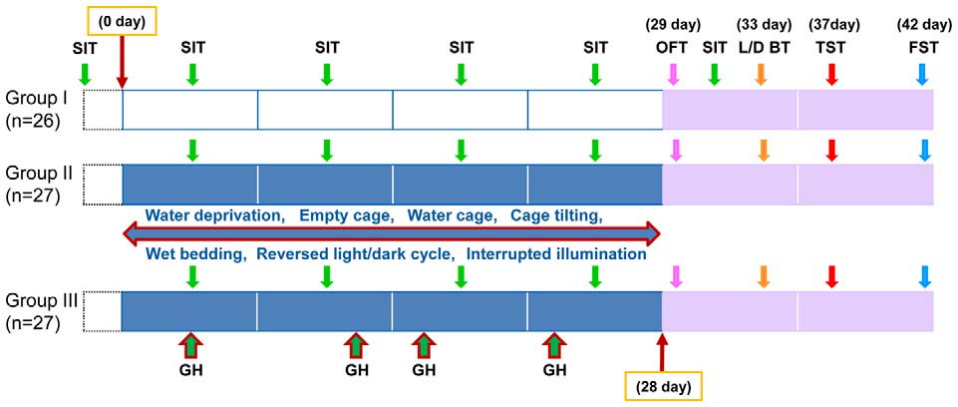
Experimental protocol. CMS procedures were performed on Group II and Group III animals for 4-weeks. The sucrose intake test (SIT) was performed at baseline, 3, 10, 17, and 24 days after CMS. The open field test (OFT) was performed at day 29. The light/dark box test (L/D BT) was performed at day 33. The tail suspension test (TST) was performed at day 37. The forced swimming test (FST) was performed at day 42. GH: Grouped housing (aggressive behavior by physical contact between two isolated mice).

The purpose of this study was to investigate whether isolation-induced aggression by physical contact could affect the anxiety and depressive-like behavior induced by the CMS model, in socially isolated male mice. In this study, we used three groups of isolated adult mice, non-stressed controls (Group I), CMS treated isolated adult mice (Group II) and CMS treated isolated mice, subjected to isolation-induced aggression by physical contact (Group III). Experimental protocol and behavioral evaluations for this study are shown in the Fig. 1.

## Results

### Sucrose consumption test

**Figure 2:**
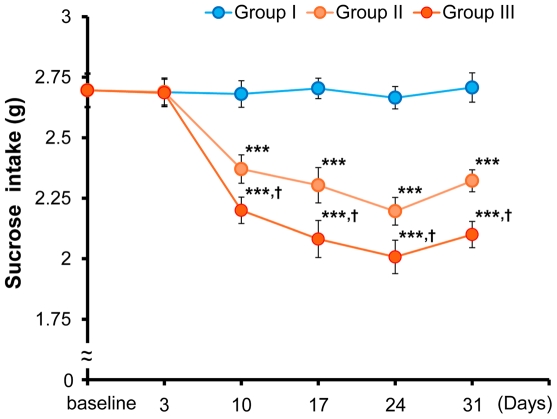
Sucrose intake test. Sucrose intake test was performed at baseline, 3 days, 10 days, 17 days, and 24 days after the start of CMS as shown in Fig. 1. The intake of 1% sucrose solution in Group II and Group III mice was significantly lower than in Group I mice at 10, 17 and 24 days after the start of CMS. Values represent the mean ± SEM (n = 26 for Group I, n = 27 for Group II, n = 27 for Group III). ***p<0.001 as compared to Group I. ^+^p<0.05 as compared to Group II.

Repeated ANOVA analysis revealed that the intake of 1% sucrose solution was significantly different (F [10,385] = 10.13, p<0.001) in the three groups (Fig. 2). One-way ANOVA showed that the intake of 1% sucrose solution was significantly different (F [2,77] = 9.19, p<0.001, F [2,77] = 10.58, p<0.001, F [2,77] = 14.07, p<0.001, F [2,77] = 16.13, p<0.001) in the three groups at 10, 17, 24 and 31 days respectively, after the start of CMS (Fig. 2). Furthermore, *post hoc* Fisher's PLSD test showed that the intake of 1% sucrose solution in Groups II and III was significantly (p<0.001) lower than in Group I mice at 10, 17, 24 and 31 days (Fig. 2). Moreover, there was a significant difference between Group II and Group III mice at 10, 17, 24 and 31 days (Fig. 2). These findings suggest that aggressive behavior induced by the physical contact of socially isolated mice could potentiate the severity of anhedonia evoked by CMS.

### Open field test

**Figure 3:**
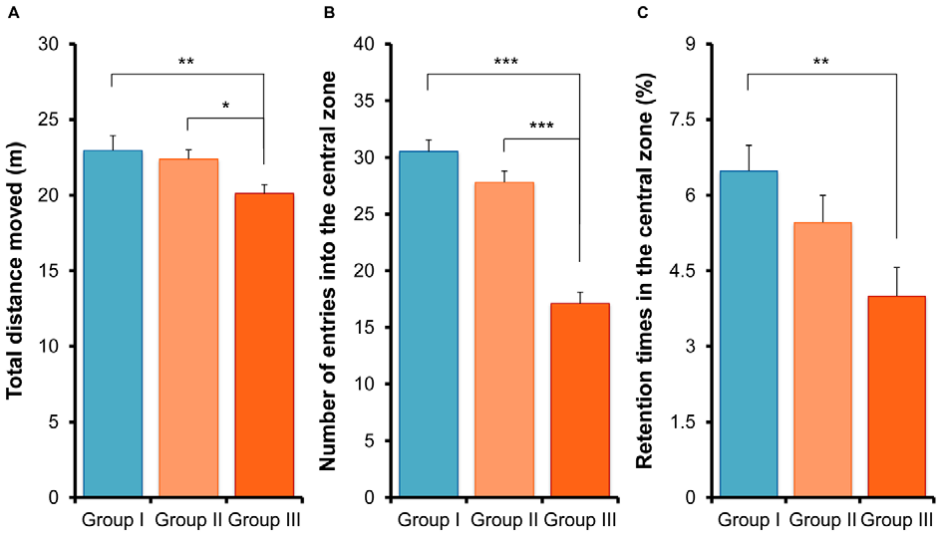
Open field test. (A): Total distance moved in 1 hour. The total distance moved by Group III mice was significantly lower than Groups I and II mice. (B): Number of entries into the central zone. The number of entries into the central zone was significantly lower in Group III compared to Groups I and II mice. (C): Retention time in the central zone. The time spent in the central zone by Group III mice was significantly lower than Group I mice. Values represent the mean ± SEM (n = 26 for Group I, n = 27 for Group II, n = 27 for Group III). *p<0.05, **p<0.01, ***p<0.001.

In the open field test, one-way ANOVA analysis revealed that the total distance moved in 1 hour was significantly different (F [2,77] = 4.27, p = 0.017) in the three groups. *Post hoc* Fisher's PLSD test showed that the total distance moved by Group III mice was significantly less (p = 0.007) than that of Group I mice (Fig. 3A), suggesting reduced locomotor activity in Group III animals. Furthermore, the total distance moved in Group III was significantly less (p = 0.029) than in Group II (Fig. 3A). However, there were no differences between Groups I and II (Fig. 3A).

One-way ANOVA analysis revealed that the number of entries into the central zone was significantly different (F [2,77] = 13.54, p<0.001) in the three groups. *Post hoc* Fisher's PLSD test showed that the number of entries into the central zone in Group III was significantly lower (p<0.001) than in Groups I and II (Fig. 3B). Furthermore, one-way ANOVA analysis revealed that the retention time spent in the central zone by the three groups was significantly different (F [2,77] = 5.28, p = 0.007). *Post hoc* Fisher's PLSD test showed that Group III mice spent significantly less time (p = 0.002) in the central zone relative to Group I mice (Fig. 3C). Moreover, animals in Group III spent less time in the central zone than those in Group II (Fig. 3C), although the difference was not statistically significant (p = 0.059). These findings suggest that in the open field test, mice in Group III, show reduced locomotor activity and anxiety-like behavior in contrast to mice in Group II.

**Figure 4:**
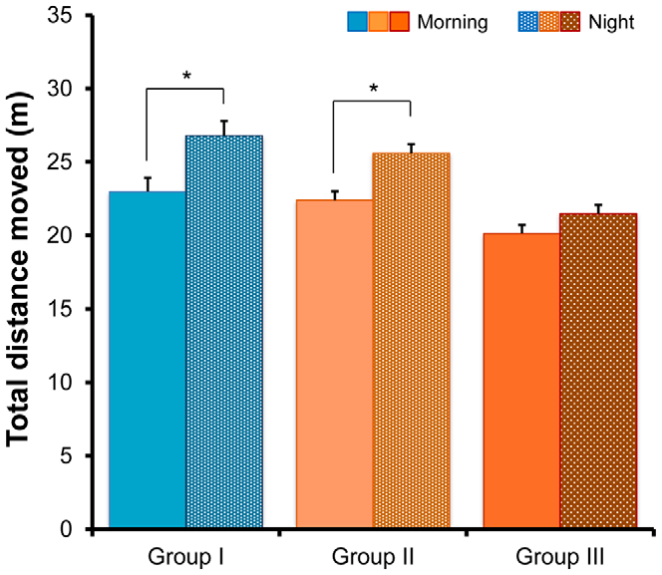
Circadian rhythm in the open field test. The total distance moved in 1 hour was measured between the hours of 9 and 10 in the morning and at night. Night time locomotor activity in Groups I and II, but not Group III mice, was significantly higher than morning activity. Values represent the mean ± SEM (n = 26 for Group I, n = 27 for Group II, n = 27 for Group III). *p<0.05.

Next, we examined circadian effects on locomotor activity in the three groups. Multivariate analysis of variance (MANOVA) revealed that there are significant effects (Wilks lambda = 3.72, p<0.006). The total distance moved in 1 hour at night by Groups I and II mice was significantly greater (Group I: p = 0.011, Group II: p = 0.21) than the distance moved in the morning (Fig. 4). Data of Fig. 4 (morning column) were same to the Fig. 3A. In contrast, the distances moved in 1 hour by Group III mice did not differ between night and morning (p = 0.311) (Fig. 4).

### Light/dark box test

**Figure 5:**
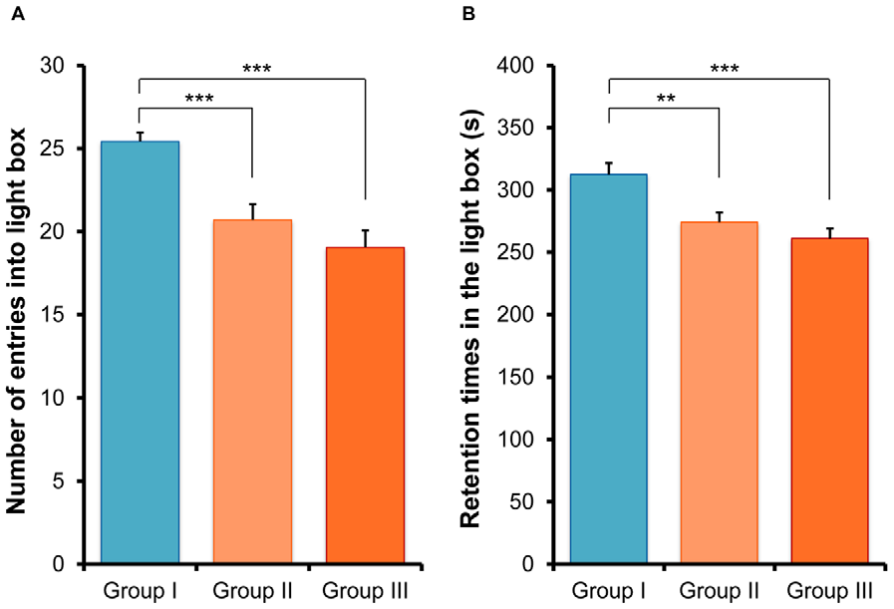
Light/dark box test. (A) Number of entries into the light box. (B) Retention time in the light box. The number of entries into the light box and the retention time in the light box for Groups II and III mice were significantly lower than for Group I mice. Values represent the mean ± SEM (n = 26 for Group I, n = 27 for Group II, n = 27 for Group III). **p<0.01, ***p<0.001.

In the light/dark box test, one-way ANOVA analysis revealed that the number of light-dark box transitions in the three groups was significantly different (F [2,77] = 14.35, p<0.001). *Post hoc* Fisher's PLSD test showed that the number of transitions by mice in the two CMS model groups was significantly lower (p<0.001 for Group II vs. Group I, p<0.001 for Group III vs. Group I) than in Group I mice (Fig. 5A). Furthermore, one-way ANOVA analysis revealed that the retention time spent in the light box was significantly different (F [2,77] = 10.29, p<0.001) amongst the three groups. *Post hoc* Fisher's PLSD test showed that the time spent in the light box by Groups II and III animals was significantly lower (p = 0.002 for Group II vs. Group I, p<0.001 for Group III vs. Group I) than Group I animals (Fig. 5B). However, there was no statistical difference between Groups II and III. These results show that in the light/dark box test, mice in Groups II and III show anxiety-like behaviors.

### Tail suspension test (TST)

**Figure 6:**
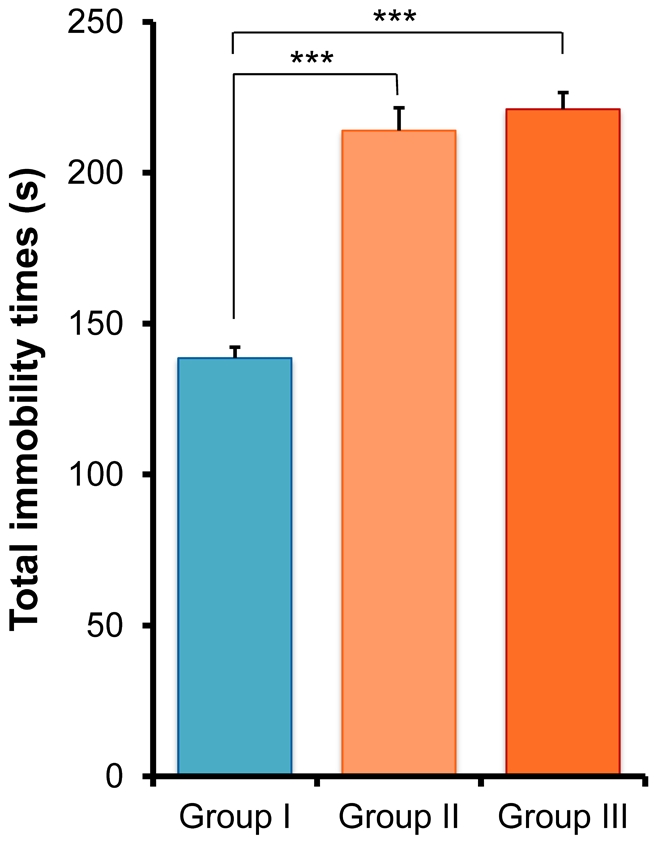
Tail suspension test (TST). The total immobility time for the two CMS model mice (Group II and Group III) was significantly longer than for controls (Group I). Values represent the mean ± SEM (n = 26 for Group I, n = 27 for Group II, n = 27 for Group III). ***p<0.001.

In the TST, one-way ANOVA analysis revealed that the immobility times were significantly different (F [2,77] = 61.19, p<0.001) in the three groups. *Post hoc* Fisher's PLSD test showed that the immobility times for Group II and Group III mice under the CMS protocol were significantly lower (p<0.001) than those for Group I mice (Fig. 6). However, there was no difference between Group II and Group III animals (Fig. 6).

### Forced swimming test (FST)

**Figure 7:**
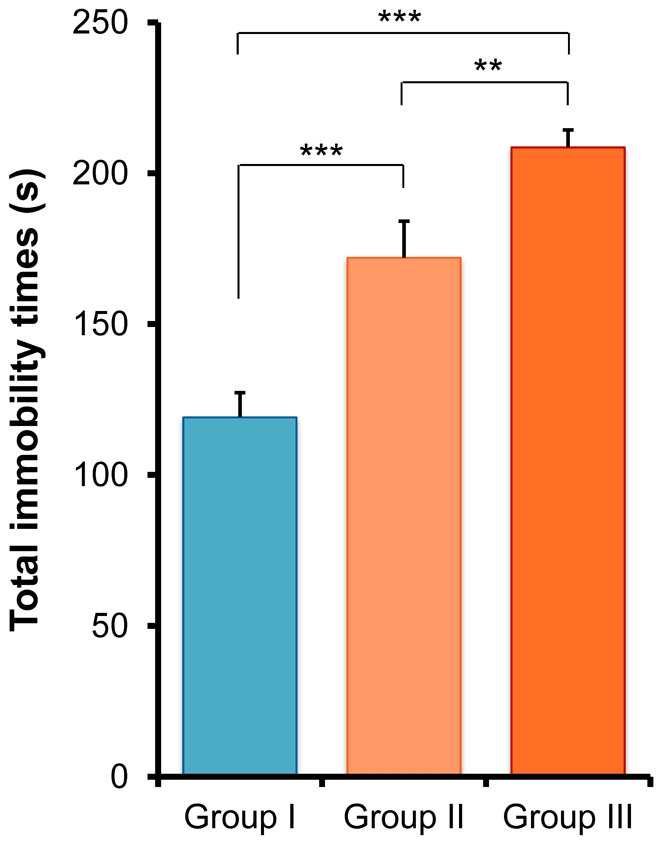
Forced swimming test (FST). The total immobility time for the two CMS model mice (Group II and Group III) was significantly longer than for controls (Group I). Furthermore, the total immobility time for Group III mice was significantly longer than for Group II mice. Values represent the mean ± SEM (n = 26 for Group I, n = 27 for Group II, n = 27 for Group III). **p<0.01, ***p<0.001.

In the FST, one-way ANOVA analysis revealed that the immobility times were significantly different (F [2,77] = 24.49, p<0.001) in the three groups. *Post hoc* Fisher's PLSD test showed that the immobility times for Group II and Group III mice were significantly (p<0.001) longer than those for Group I mice (Fig. 7). Interestingly, there was a significant difference (p = 0.005) between Group II and Group III animals (Fig. 7).

## Discussion

The major findings of this study are that the unpredictable CMS procedure induced anxiety and depressive-like behavior in male adult mice with social isolation, and that aggression by physical contact in isolated mice could potentiate anxiety and depressive-like behavior. In the CMS paradigm, social interaction stress (such as, putting together in the same cage, two mice that have previously been housed separately) has been widely used, although animals usually do not show aggressive behavior [25]. In this study, we used aggression by physical contact between two previously isolated mice as a stressor in the CMS paradigm. Therefore, the present method is clearly distinct from the widely used CMS paradigm. To our knowledge, this is the first paper showing that social isolation-induced aggressive behavior might potentiate anxiety and depressive-like behavior caused by CMS of socially isolated mice.

In the sucrose intake test, the CMS paradigm produced a decrease of 1% sucrose intake, consistent with previous reports [34]–[36]. Here we found that aggression by physical contact in isolated mice potentiated the decrease of 1% sucrose consumption in isolated mice within the CMS model, showing that social isolation-induced aggressive behavior may increase the severity of anhedonia in isolated male mice.

In the open field test, the locomotor activity of Group III mice was significantly decreased compared with Group I animals. Furthermore, locomotor activity of Group III mice was significantly lower than in Group II, suggesting that aggression by physical contact in isolated mice might decrease locomotor activity of mice within the CMS paradigm. Moreover, mice in Group III visited less frequently and spent less time in the central zone of the open fields compared with Group I. In addition, the number of entries into the central zone by Group III mice was significantly lower than in Group II mice, suggesting that aggression by physical contact of isolated mice might exacerbate anxiety-like behavior. We found that the locomotor activity of mice in Group III did not differ between morning and night, suggesting that aggression by physical contact of isolated mice may disrupt circadian activity in the mice within the CMS model. Since patients suffering from depression also experience a wide of range of circadian rhythm disturbances [37], [38], this finding may be of interest. To confirm the abnormalities of circadian rhythm in the Group III mice, it would be necessary to measure locomotor activity for a longer period of time on the accustomed apparatus.

In the light/dark box test, the number of entries and the time spent in the light box by animals in Groups II and III were significantly reduced compared with control mice in Group I, although there was no difference between Groups II and III animals. These findings indicate that the CMS paradigm may cause anxiety-like behavior in these socially isolated male mice.

In the TST and FST, the immobility times of mice in Group II and Group III were significantly longer than in controls (Group I), which is consistent with previous reports [20], [39]–[43]. Interestingly, we found that isolated mice exposed to aggression by physical contact showed significantly increased immobility times in the FST, whereas the immobility times in the TST remained the same. Although the reasons underlying this difference are currently unclear, it is likely that the neurobiological pathways mediated by these two models are different [44]. For example, quantitative trait loci (QTL) analysis using C57/B6 mice identified genes that may contribute to the difference responses in immobility times between the TST and FST [45]. This highlights the genetic contribution to the behavioral performances in these two paradigms. Nonetheless, it should be noted that isolation-induced aggressive behavior could increase depressive-like behavior in isolated male mice subjected to unpredictable CMS.

It is well known that social isolation of male mice induces offensive aggressive behavior [31], [33]. A number of neurotransmitters, including serotonin, norepinephrine, dopamine, and GABA, and BDNF are thought to be involved in the is social isolation –induced aggression [46], [47]. It has been reported that early social isolation in mice induces robust changes in postsynaptic, serotonergic receptor gene transcription, motor hyperactivity and behavioral disinhibition [48]. Furthermore, serotonergic drugs, including selective serotonin reuptake inhibitors, reverse isolation-induced aggressive behavior in male mice, suggesting a role for serotonergic neurotransmission in isolation-induced aggression in male mice [49], [50]. It is therefore likely that disturbances in serotonergic neurotransmission may be observed in the brain of our CMS model mice.

The CMS models are considered to be of high face, construct and predictive validity. In these models, prolonged exposure to uncontrollable and unpredictable stressors results in depressive-like behavior that can be prevented or reversed by chronic but not acute antidepressant treatment [20], [25], [26]. Very recently, Li et al. [51] reported that a single administration of the *N*-methyl-D-aspartate (NMDA) receptor antagonist ketamine produced rapid antidepressant effects in rat CMS models. Given the role of glutamate in the rapid antidepressant action of ketamine [12], [52]–[55], it may be of interest to examine the effects of ketamine within our CMS model.

In conclusion, this study suggests that aggressive behavior evoked by physical contact in isolated mice could potentiate anxiety and depressive-like behavior in adult male mice subjected to unpredictable CMS. Therefore, this CMS model may be a useful animal model of depression.

## Materials and Methods

### Animals

Eighty adult male C57 BL/6J mice (age: 7±1 weeks; average body weight: 20±2 g) were purchased from the Experimental Animal Center of Shaanxi Province (Xi'an, PR China). Mice were housed singly in cages (26 cm×18 cm×13 cm) under a controlled 12-hour/12-hour light-dark cycle (lights on: 7:00 a.m.), with a room temperature of 21±2°C and humidity of 55±5%. Mice were given free access to water and food. The experimental protocols (Permit Number: 200910011) were approved by the Xi'an Jiaotong University Laboratory Animal Administration Committee and performed according to the Xi'an Jiaotong University Guidelines for Animal Experimentation and also conformed to the Guide for the Care and Use of Laboratory Animals published by the US National Institutes of Health. All efforts were made to minimize suffering.

Mice were allowed to adapt to the stable environmental conditions for 1 week, and then a baseline of 1% sucrose solution consumption was measured for 3 weeks, 3 times per week (on Mondays, Wednesdays and Fridays), for a period of 1 hour during the hours of 9:00–10:00 a.m.. When a stable baseline of sucrose consumption was achieved, mice were divided into 3 groups. Twenty six mice were assigned as non-stressed controls in, Group I, 27 mice to Group II and subjected to CMS procedures and 27 mice were assigned to a Group III and subjected to CMS procedures and aggressive behavior by the physical contact of two normally isolated mice (Fig. 1). There were no significant baseline differences in sucrose consumption and body weight amongst the animals.

### CMS paradigm

The mice in Groups II and III of the CMS model received a variety of stress, including 45°cage tilting, cage-switching, empty cage, soiled cage, empty cage with water on the bottom, continuous overnight illumination, inversion of the light/dark cycle. These stresses were applied randomly, during both light and dark periods (Fig. 1). Mice in Group III were paired randomly with each other and housed together for 2 hours, once a week. These mice showed aggressive behavior (e.g., biting attack, lateral threat, aggression and tail rattle) when two isolated mice were placed in the same cage. The CMS paradigm procedures spanned a 4 week period (Fig. 1).

### Sucrose intake test

After the CMS procedure was started, 1% sucrose intake was measured between 9:00–10:00 a.m. every Wednesday for 1 hour (Fig. 1). Fourteen hours before the sucrose intake test, all mice (including the control group) were deprived of water and food and all CMS procedures were halted. Mice resumed eating and drinking freely after the sucrose intake test. Control mice in Group I were kept under the same laboratory conditions, in a different room.

### Other behavioral tests

Behavioral tests were performed in the following order: open-field test on the 1^st^ day, light/dark box test on the 5^th^ day, tail suspension test (TST) on the 9^th^ day and forced swimming test (FST) on the 14^th^ day (Fig. 1). Mice were put into the test room 30 minutes before the test. All tests were performed between 9:00–10:00 a.m. in a quiet room. After each test, mice were replaced in their individual cages and returned to breeding room.

### Open-field test

According to previous methods [56], the apparatus consisted of a square box with dimensions, 45 cm×45 cm×45 cm. Mice were placed into the center of the open box under a dark light (25 lx) and allowed to explore the arena for 1 hour between the hours of 9:00–10:00 a.m. and 9:00–10:00 p.m. A video-computerized tracking system (SMART, Panlab SL, Barcelona, Spain) was used to record the distance traveled as a measure of locomotor activity.

### Light/dark box test

The dark/light box consisted of two equal sized metal compartments (15 cm×16 cm×18 cm), one dark and one illuminated by light of 50 lx intensity, connected by a tunnel. Mice were placed into the dark compartment, from where they could visit the lit box. The total duration of time spent in the light box and the number of visits to this anxiety-related compartment were scored by visual observation for 6 minutes.

### Tail suspension test (TST)

Mice tails were wrapped with tape from base to tip, covering about 4 / 5 of its length and fixed upside down on the hook. The immobility time of each mouse was recorded over a 6 minute period. Mice were considered immobile only when they hung passively and completely motionless. Mouse groups were blinded to observer assessing immobility.

### Forced swimming test (FST)

Equipment for this test consisted of a glass barrel (high×diameter: 25 cm×15 cm) with 10 cm of water at room temperature (about 22±1°C). A mouse was placed in this barrel and immobility time was measured for 6 minutes using a video surveillance system (SMART, Panlab SL, Barcelona, Spain). After testing, the mice were removed into a normal heat preservation breeding cage with padding and covered with an absorbent towel. The cage was then placed in an electric dryer at 30–35°C for about 20 minutes.

### Statistical analysis

The data are expressed as the mean ± standard error of the mean (S.E.M.), and data analysis was performed using the PASW Statistics 18 (formerly SPSS statistics; SPSS, Tokyo, Japan). The data for the sucrose intake test were analyzed by repeated measures analyses of variance (ANOVA), and one-way ANOVA followed by *post hoc* Fisher's PLSD test. Behavioral data, including open field test, light/dark box test, TST, and FST, were analyzed by one-way ANOVA, followed by *post hoc* Fisher's PLSD test. The open field test in morning and night data were analyzed by multivariate analysis of variance (MANOVA), followed by Student's t-test. *P* values of less than 0.05 were considered statistically significant.
